# Active play in ASP –a matched-pair cluster-randomized trial investigating the effectiveness of an intervention in after-school programs for supporting children’s physical activity

**DOI:** 10.1186/s12889-020-08645-1

**Published:** 2020-04-15

**Authors:** Kirsti Riiser, Kåre Rønn Richardsen, Anders L. H. Haugen, Siv Lund, Knut Løndal

**Affiliations:** 1 Department of Physiotherapy, Faculty of Health Sciences, OsloMet - Oslo Metropolitan University, PO Box 4, St. Olavs plass, N-0130 Oslo, Norway; 2 Department of Primary and Secondary Teacher Education, Faculty of Education and International Studies, OsloMet - Oslo Metropolitan University, PO Box 4, St. Olavs plass, N-0130 Oslo, Norway

**Keywords:** After-school programs, Physical activity, Sedentary behavior, Physical activity play, Self-determination theory, Activity support, Accelerometer

## Abstract

**Background:**

Interventions directed at after school programs (ASPs) have the potential to support physical activity (PA) in young children. Research has indicated that interventions that emphasize competence building among the ASP staff can lead to increased PA among the children. The present study evaluates the effectiveness of the *Active Play in ASP* intervention—a program for ASP staff aimed at supporting physical activity among first graders in ASP.

**Methods:**

We used a matched-pair cluster randomized design and included 456 first graders from 14 schools in Norway. From these, 7 ASPs received the intervention (*N* = 229), while 7 acted as controls (*N* = 227). Measurements were taken at baseline, immediately post intervention (7 month follow-up) and after a year (19 month follow-up). The primary outcome was moderate to vigorous physical activity (MVPA), which was estimated with predefined cut points of counts per minute (CPM) and expressed as minutes/hour. Secondary outcomes were vigorous and light intensity physical activity (VPA and LPA) and sedentary behavior. The analyses of intervention effects were based on between-group differences in outcome changes between the 3 measurement points and were conducted using a mixed-effects model for repeated measures using categorical time. In exploratory analyses, we investigated gender, baseline body mass index, and baseline CPM as potential effect modifiers.

**Results:**

No significant intervention effects was observed on MVPA (0.55 min/hour [99% CI -0.55:1.64]) or on the secondary outcomes, min/hour of LPA, VPA or sedentary behavior. Exploratory analyses indicated that among the 50% least physically active children at baseline, children in intervention ASPs reduced sedentary time from baseline to 19 months follow up by 1.67 min/hour (95% CI -3.12:-0.21) compared to the controls.

**Conclusions:**

Although the intervention did not significantly increase the mean MVPA among the children in the intervention ASPs compared to controls, it did seem to have a small effect by reducing sedentary behavior time among the least active children. An even stronger emphasis on how to identify less active children and support their activity may be needed in order to increase their PA and further reduce sedentary behavior time.

**Trial registration:**

ClinicalTrials; NCT02954614, Registered 3 November 2016, −Retrospectively registered, first participant enrolled August 2016

## Background

Promoting physical activity is an essential strategy for improving the health of individuals and populations [1]. Over the years, increased attention has been given to the relationship between moderate to vigorous physical activity (MVPA) and children’s health and well-being [2]. Consequently, the importance of early PA adoption by children is well documented and, in addition to its influence on numerous health factors, research shows that PA participation during childhood can predict PA levels in adulthood [3].

PA is considered to be a collective term that includes physical activity play, hiking and more organized forms of sport activities. Young schoolchildren are mainly physically active through play that includes locomotory movements, manipulative movements, and stabilizing postures [4]. In early childhood, increased PA provides opportunities for motor development while motor skill competence drives PA [5]. Adequate motor skills are needed for participation in both physical activity play and PA in general. Children with less-proficient motor skill competence levels engage in lower levels of habitual PA [6]. Research also shows that motor competence is associated with physical fitness and this correlations is particularly strong among children aged 4–6 [7, 8]. Hence, PA at high intensity levels among the children is of great importance. It is widely recommended that children engage in MVPA for at least 1 hour per day [9]. Moderate-intensity activity requires a person to work hard enough to raise one’s heart beat and break a sweat, while vigorous activity significantly increases an individual’s heart rate [1]. However, development of motor competence requires a variety of activity types at levels ranging from light to vigorous [10].

Increasing PA opportunities for children in school has emerged as a priority worldwide. Several attempts have been made to develop school-based PA interventions and new interventions are continuously being evaluated. Although many studies show promising results, the magnitude of observable effects is generally small and research on long-term impacts is needed [11]. One major challenge for PA interventions in schools is that PA possibilities are often limited to short recesses and physical education classes. Thus, children are not provided with sufficient opportunities to be physically active in school and after school programs (ASP) are vital settings for promoting PA among children. Norwegian studies have found that young schoolchildren (6 years of age) had a mean daily MVPA of nearly 30 min while staying in an ASP, however, a minority of children accumulated almost no PA at all, which is of concern [12, 13]. Moreover, there were significant gender differences—with boys having higher MVPA and less sedentary behavior in comparison to girls. All groups, the most as well as the least active, increased their PA intensity when they had to be outdoors [13].

The Norwegian ASPs are public programs for children in first to fourth grades (6–10 years of age). As many as 81% of first graders (5–6 years old) attend an ASP for 10 to 20 h each week [14]. They are organized by schools, but attendance is voluntary. The ASPs are generally located in the school area with access to the school’s equipment, facilities, and playground. In contrast to the sports-dominated extra-curricular physical activity in other countries [15], Norwegian ASPs are expected to encourage self-managed activities for the children [16, 17]. In Norway, as in many other countries, only a minority of the ASP staff have formal pedagogical education. Previous research has indicated that interventions, including competence-building measures for ASP employees, can lead to increased PA among the children [18–21]. Studies stress that ASPs should emphasize encouragement and positive feedback on the children’s physical activities, development of PA schedules, environment structuring, and PA arrangement administration. Increased competence among the ASP staff can be achieved through closer cooperation between the ASP and professionals with particular competence in planning, facilitating, and promoting physical activity play, as well as in assessing motor skills and difficulties in children. School physiotherapists, who play an essential role in the delivery of primary healthcare to children and adolescents in Norway, and trained physical education teachers are both skilled professionals who could be integrated further into ASPs.

No previous research exists on systematically developed, theory-based PA-interventions in Scandinavian ASPs. Moreover, there is a general lack of studies that specifically investigate PA in young child populations. Hence, the objective of this study was to evaluate the effectiveness of the *Active Play in ASP* intervention—a course program for ASP staff aimed at supporting physical activity play and PA among first graders in ASPs. We hypothesized that this intervention would have immediate and long-term beneficial effects on children’s PA in terms of increased time spent in MVPA, vigorous PA and light PA and less time spent in sedentary behavior.

## Methods

### Design

This study has previously been described in detail elsewhere [22]. It followed the recommendations of the CONSORT statement and was registered with ClinicalTrials.gov (registration number NCT02954614). A matched-pair cluster-randomized controlled design was applied, with ASPs as clusters and first grade pupils as participants. The ASPs in the intervention group received the *Active Play in ASP* intervention, while the ASPs in the control group were given no follow-up. The ASPs randomized to control were invited to receive the intervention after the study was completed.

### Recruitment and sample

**Fig. 1:**
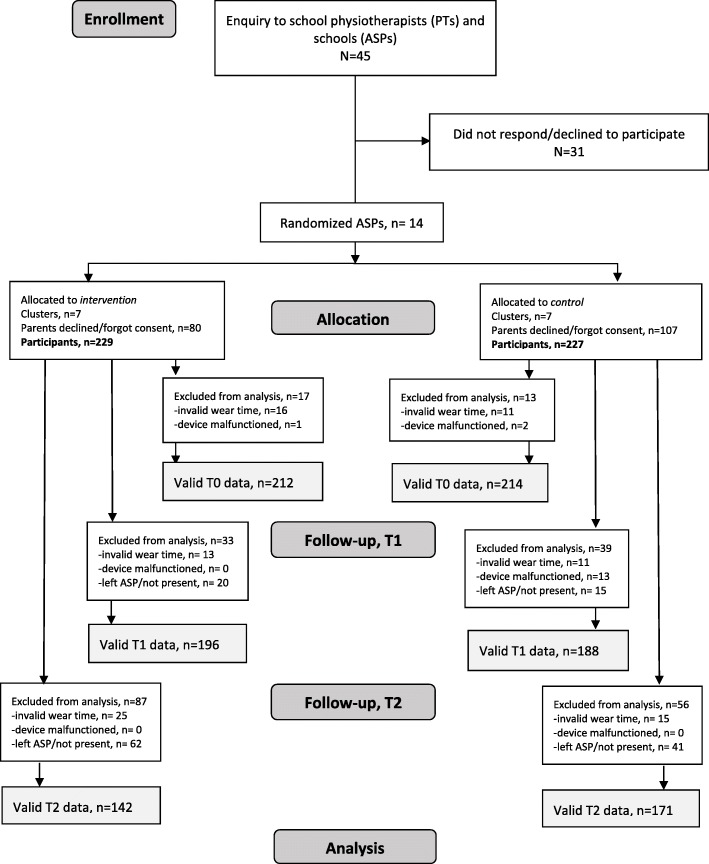
The flow of the participants in the study

The first step of the study recruitment process was to engage school physiotherapists (PTs) because the study relied on their assistance in the implementation of the intervention as well as in the data collection process. School health services in municipalities of three counties in Eastern Norway were approached and, within the time limit defined for this first phase of recruitment (August 2016), PTs from 14 municipalities volunteered to participate. They assisted in recruiting the ASPs in schools within their area of responsibility. All schools were eligible. School administrators, who accepted the invitation, provided written consent. The ASPs were paired based on available background information about the size (small, medium, large) and location (urban versus rural), as we assumed that the number of pupils, as well as space and access to nature areas, might have an impact on children’s PA level. The names of the ASPs were put in sealed envelopes and randomly allocated to either the intervention or control group. The person revealing the allocation was not involved in the study [22]. Following the allocation, all parents of first graders (5–6 years of age) in the participating ASPs were asked to provide written consent on behalf of their child. There were no exclusion criteria. First graders were chosen as study subjects because a great majority of them attend ASPs in Norway [14]. Moreover, in comparison to older children, there is less information on PA in this age group. Figure 1 shows the flow of the participants in the study. Sample size calculation was performed based on previous studies and on the pilot study, and we assumed that a 6-min increase in MVPA during ASP opening hours in the intervention compared to the control (common SD = 12) would be of clinical importance [18, 19, 22], which represents 10% of the 60 MVPA minutes recommended by the guidelines. We estimated N to be 121 in each group, without accounting for cluster effects, and planned to enroll a minimum of 200 children in each group to secure sufficient power for additional analysis at the cluster level. With a minimum of 25 first graders estimated in each ASP, a maximum of 16 ASPs would have to be included [22].

### The intervention

The *Active Play in ASP* intervention consisted of a 7-month course program (October–May) directed at ASP staff. The intention was to enhance their knowledge of and skills in creating a PA-supportive environment by accommodating and gently encouraging activities instead of directing them in a controlling manner. The intervention was informed by experiences from practice-, theory-, and evidence-based knowledge. It was developed in a stepwise process, together with representatives from practice, and was built on principles of the self-determination theory (SDT) [22, 23]. In line with the SDT principles, the intervention focused on the importance of retaining the self-chosen and child-managed character of play with an emphasis on supporting the children’s needs for competence, relatedness, and autonomy [24]. Prior to starting in the ASPs, the local PTs in the intervention group were provided with an 8-h course that included a presentation of the intervention study with an emphasis on their role and responsibility. They were also provided with intervention material (workbook). The intervention course program for the ASP staff consisted of two introductory sessions led by two of the researchers, KL (PhD and PE teacher) and KR (PhD and physiotherapist) focusing on the children’s physical activity play, the importance of friends, and the activity place. Furthermore, the sessions emphasized the ASP staff’s interaction styles, the children’s motivation for PA, and the ways in which the staff could support the children’s engagement in PA. The sessions took place in each participating intervention ASP and included short lectures, theme-based discussions, and group tasks. The staff were encouraged to provide examples from their own practical experience. Following the introductory sessions, one of the researchers met with the staff to outline how their ASP would combine new knowledge and previous experience into strategies for supporting children’s physical activity play during the time they spend at that ASP. The local PT participated in the introductory sessions and the meeting with researchers. Additionally, the local PT and an ASP representative were responsible for mapping the activity equipment as well as the indoor and outdoor facilities available at each ASP. This information provided an important supplement to the discussions that followed. The program continued during the school year, with monthly meetings for the ASP staff led by the local PT during which they worked together on predefined tasks related to physical activity play (Table 1).

**Table 1:** Intervention components and content

	Component	Content
**Introductory course for school physiotherapists**	1-day course	Information about the intervention and the physiotherapists’ role and responsibilities. Presentation of intervention workbook.
**Course program for ASP staff**	3-h session	Introduction to research-based knowledge on children’s physical activity in play. Increasing the staff’s awareness of how such play can be influenced and supported in ASPs
	3-h session	Basic theoretical principles of SDT applied to physical activity and physical activity play among children; how to be activity supportive.
	Mapping	Thorough mapping of the ASP equipment and facilities.
	Planning (1–2 h meeting)	Summary of introductory sessions; how to make use of new knowledge.
	5 meetings (monthly 1–2 h) led by the local school physiotherapist	Discussions and practical tasks focusing on: - Motor learning in children, - Equipment and environment, - Mapping of staff competencies, - Inclusion/exclusion in play, - Leading and supporting activity in groups.

The table has previously been published in the protocol article [22]. No permissions were required to include it here

### Outcome measures

The data were collected at 3 time points—in September/October 2016 (before the beginning of the intervention; baseline, T0), 7 months later (immediately post intervention; follow-up 1, T1), and 1 year post intervention (follow-up 2; T2). All outcomes pertained to individual participants. The primary outcome was MVPA and secondary outcomes were VPA, LPA, and sedentary behavior. PA was measured during the time spent in an ASP during its opening hours for each ASP on weekdays over a period of 1 week. Objective PA assessments were obtained using the ActiGraph GT3X (Actigraph™ LLC, Pensacola, US). The ASP staff administered the children’s accelerometers following a standard procedure. The devices were fitted to each child’s hip at the time of arrival and removed before they left. Valid accelerometer data files consisted of at least 2 days of minimum 60 min of accelerometer wear time. All data registered before or after each ASP’s opening hours were regarded as non-wear time and were excluded from the analysis. Non-wear time was defined as 20 min of consecutive zeros, allowing for 2 min above zero. ActiLife version 6.13.3 was used to download raw accelerometer files and to reintegrate them in 10 s epochs to detect the intermittent and sporadic activity patterns of children. Subsequently, the files were loaded into Kinesoft v3.3.80 (Kinesoft, SK) for screening and classification of intensity level. The number of minutes spent in MVPA was estimated using cut points, as described and recommended in previous studies [25, 26]. MVPA was defined as 2296–4011 counts per minute (CPM), while > 4011 CPM was defined as VPA, 101–2295 CPM as LPA, and < 101 CPM as sedentary behavior [25].

The local PTs measured the participating children’s height and weight using a stadiometer and a digital scale at the local school nurses’ office. The children wore light clothes and no shoes. The Body Mass Index (BMI = kg/m^2^) was calculated and converted into age and gender specific scores [27]. The children’s gender was reported by the parents at the beginning of the study.

The data on average weekly outdoor temperature (degrees Celsius) were calculated for each school at baseline, follow-up 1, and follow-up 2, based on the daily mean temperatures that were estimated using the data recorded at local weather stations closest to each ASP during the ASP opening hours (at 14:00 PM). The average rainfall (in mm) was also documented for each ASP (between 12:00 PM and 17:00 PM).

### Statistical analyses

Descriptive statistics were generated for the gender and BMI category variables for each group. The analyses of intervention effects were based on the between-group differences in MVPA (primary outcome), VPA, LPA and sedentary behavior (secondary outcomes) changes between T0, T1, and T2, respectively. There was a large variance in the participants’ average daily wear time at each measurement point. Thus, wear time would influence the MVPA minutes accumulated per day. To account for the variance in daily wear time, MVPA, VPA, LPA, and sedentary behavior were expressed as minutes/hour.

To account for clustering, we included random effects for schools and for pupils nested within schools. The analyses were conducted using mixed-effects models, and we employed the longitudinal data analysis (LDA) method to adjust for baseline value [28]. In line with this method we included treatment, time (categorical), and their two-way interaction term as fixed factors in the main effects analyses of the pre-specified analysis. We controlled for the daily mean outdoor temperature at each school. We used an unstructured covariance pattern for level 1 residuals (or autoregressive patter if non-convergence), and an identity covariance structure for random effects [28].

Tests of effect modification were not specified in the protocol, and thus treated as exploratory [29]. In separate models, we investigated gender, baseline BMI and baseline accelerometer counts per minute (CPM) as potential effect modifiers at each time point through inclusion in the three-way interaction terms (treatment x time x “effect modifier”) [30]. We explored differential intervention effects for children with high vs. low baseline BMI using a binary measure; For the analyses, children with overweight and obesity (age- and gender adjusted BMI ≥ 25) were collapsed into one category. We used a binary measure of baseline CPM (CPM < median = 50% least physically active and CPM ≥ median = 50% most physically active) to explore the differential intervention effects for children with high vs. low levels of baseline physical activity.

As specified [22] the main analyses were conducted using an alpha level of 0.01, while in the exploratory analyses the alpha level was set at 0.05. Statistical analyses were performed using STATA 15.0 [31]. The LDA method used all available participant data and were based on the missing at random (MAR) assumption [28, 32]. We conducted multiple imputation by chained equations (MICE) as a sensitivity analysis [33]. In addition to the variables in the analytic models, the auxiliary variables included in the imputation model were baseline BMI (continuous) and gender. In order to take into account the three-level structure of the data in the imputation model, we used the “ml.lmer” command from the R package “miceadds” R. We generated 25 imputed datasets and used the “lmer” command from the “lme4” R package to analyze them. The imputation procedure was performed using the RStudio version 1.1.423.

### Ethical considerations

The study was reviewed and approved by the Data Protection Official for Research to ensure that the project was conducted in accordance with the Personal Data Act and the Personal Health Data Filing System Act (reference number 46008). Informed written consent to participate was obtained from the parents on behalf of their children. Adjusted oral information was given to the participating children at each measurement point.

## Results

Altogether, 14 ASPs agreed to participate and to be randomized into either the intervention or control group. Parents of 456 children consented to have their child participate in the study, while parents of 187 children either did not provide written consent or rejected consenting to participation. Missing data at each time point occurred due to malfunctioning accelerometers, insufficient wear time, a participating child not being present at the ASP, or having permanently quit the ASP (Fig. 1). Consequently, 426, 383, and 313 participants were included in the analyses at baseline, follow-up 1, and follow-up 2, respectively. At baseline, 47.8% of the participants were female and 14.9% of the participants were overweight or obese (Table 2).

**Table 2:** Description of participants attending the Active Play in ASP study

	Consenting children^**a**^ ***N*** = 456	Participating children^**b**^					
		Baseline (T0) ***N*** = 426		Follow-up 1 (T1) ***N*** = 383		Follow-up 2 (T2) ***N*** = 313	
		Intervention *N* = 212	Control *N* = 214	Intervention *N* = 199	Control *N* = 184	Intervention *N* = 142	Control *N* = 171
Gender, n (%)							
Girls	218 (47.8)	97 (45.8)	105 (49.1)	99 (49.7)	91 (49.5)	70 (49.3)	93 (54.4)
Boys	238 (52.2)	115 (54.2)	109 (50.9)	100 (50.3)	93 (50.5)	72 (50.7)	78 (45.6)
BMI, n (%)							
BMI < 25	350 (76.8)	182 (85.8)	168 (78.5)	167 (83.9)	139 (75.5)	115 (81.0)	127 (74.3)
BMI ≥25	68 (14.9)	28 (13.2)	40 (18.7)	28 (14.1)	27 (14.7)	26 (18.3)	31 (18.1)
Missing	38 (8.3)	2 (0.9)	6 (2.8)	4 (2.0)	18 (9.8)	1 (0.7)	13 (7.6)

^a^Enrolled in the study following parental consent

^b^Participating children providing valid accelerometer data at each data collection period

In both the intervention and control groups, the mean number of monitoring days was 4.2 days at baseline, while the mean total wear time in minutes/week was 682.2 and 707.1 min, respectively. There were no statistically significant differences in the mean activity levels between the intervention and the control group. The mean wear time declined from baseline to follow-up 1 and follow-up 2 for both groups (Table 3). The average weekly temperature (C°) varied across schools and across timepoints (Table 3). The average precipitation did not vary between the school clusters and was thus not adjusted for in the analyses.

**Table 3:** Description of the accelerometer wear time, the PA and the sedentary behavior time at each measurement

	Baseline (T0) ***n*** = 426		Follow-up 1 (T1) ***n*** = 383		Follow-up 2 (T2) ***n*** = 313	
	Intervention ***N*** = 212	Control ***N*** = 214	Intervention ***N*** = 199	Control ***N*** = 184	Intervention ***N*** = 142	Control ***N*** = 171
**Accelerometer wear, mean (SD)**						
Total weekly wear time (minutes)	682.2 (205.9)	707.1 (208.3)	642.9 (212.9)	659.4 (225.9)	559.5 (227.4)	614.2 (191.1)
Total wearing days	4.2 (1.0)	4.2 (0.9)	4.0 (1.1)	3.9 (1.1)	3.7 (1.1)	3.8 (1.0)
Daily wear time (minutes)	160.8 (29.8)	168.1 (31.7)	159.7 (34.1)	165.8 (28.6)	150.6 (33.8)	160.6 (32.3)
**Physical activity, mean (SD)**						
MVPA min/hour	10.0 (4.0)	9.0 (3.3)	9.2 (3.6)	9.8 (4.3)	10.5 (4.7)	9.2 (3.4)
VPA min/hour	3.8 (2.1)	3.4 (1.7)	3.4 (1.8)	3.8 (2.3)	3.9 (2.6)	3.7 (1.9)
Light PA min/hour	26.7 (3.3)	25.9 (3.4)	26.5 (3.1)	26.3 (3.3)	26.4 (2.9)	25.9 (3.6)
Sedentary min/hour	23.3 (5.6)	25.2 (5.2)	24.4 (5.1)	24.0 (5.3)	23.1 (5.6)	24.8 (5.3)
CPM	1083.11 (358.56)	1101.42 (301.44)	1026.58 (330.18)	1077.33 (372.12)	1152.24 (450.29)	1088.9 (352.94)
**Weather, mean (SD)**						
Average weekly temperatue (C°)	6.9 (2.9)	6.4 (1.4)	10.5 (4.8)	14.2 (5.3)	9.5 (1.6)	9.8 (2.1)

### Primary outcome

**Fig. 2:**
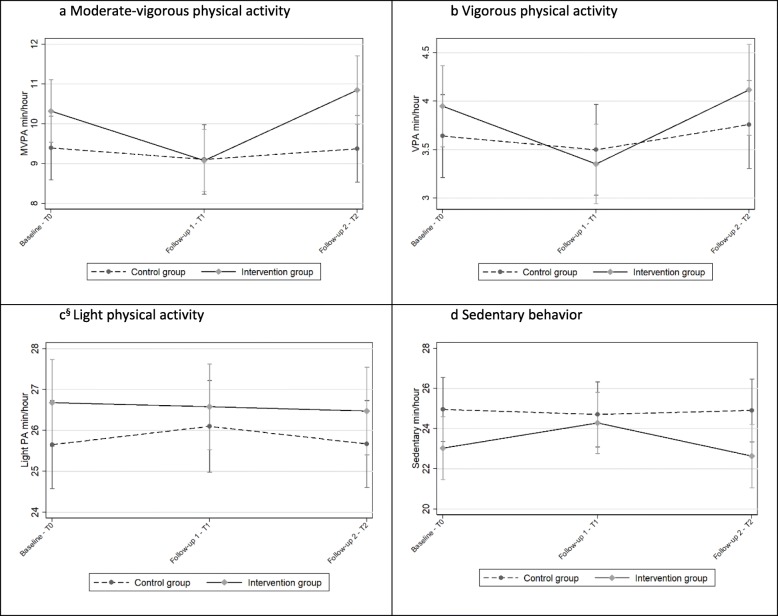
**a–d**: Trajectories showing the mean MVPA, VPA, LPA, and sedentary behavior min/hour for both groups. T0: *N* = 426, T1: *N* = 383, T2: *N* = 313. §Modelled with an autoregressive (AR 1) residual covariance matrix because the unstructured covariance matrix did not converge

The main effect analyses showed no statistically significant positive effect on MVPA min/hour in the intervention group compared to the control group. However, a statistically non-significant negative change in MVPA min/hour between baseline and follow-up 1 was observed in the intervention group contrasted with the control group (− 0.95 MVPA min/hour [99% CI -1.93: − 0.30]) (Fig. 2a). From baseline to follow-up 2, a non-significant positive change in MVPA min/hour was seen in the intervention group compared to the controls (0.55 MVPA min/hour [99% CI -0.55:1.64]) (Fig. 2a).

### Secondary outcomes

There were also no intervention effects on the secondary outcomes. A decreasing trend in VPA from baseline to follow-up 1 was observed in the intervention group in contrast to the control group (− 0.45 VPA min/hour [99% CI -1.02: 0.11]). There was no statistically significant difference between the groups in terms of VPA change between baseline and follow-up 2 (Fig. 2b). There were no significant differences between the intervention and control groups regarding changes in LPA (Fig. 2c). Sedentary time in the intervention group increased by 1.50 min/hour (99% CI 0.18: 2.99), relative to the control group from baseline to follow-up 1; however, no change was seen between baseline and follow-up 2 (Fig. 2d).

### Exploratory analyses

The exploratory analyses of differential treatment effects on the primary (MVPA) and secondary outcomes (VPA, LPA, sedentary behavior) for girls and boys showed no significant difference in change from baseline to follow-up 2. Also, there were no significant differences in any of the trajectories for the children with an age- and gender adjusted baseline BMI of < 25 vs. a BMI of ≥25.

**Fig. 3:**
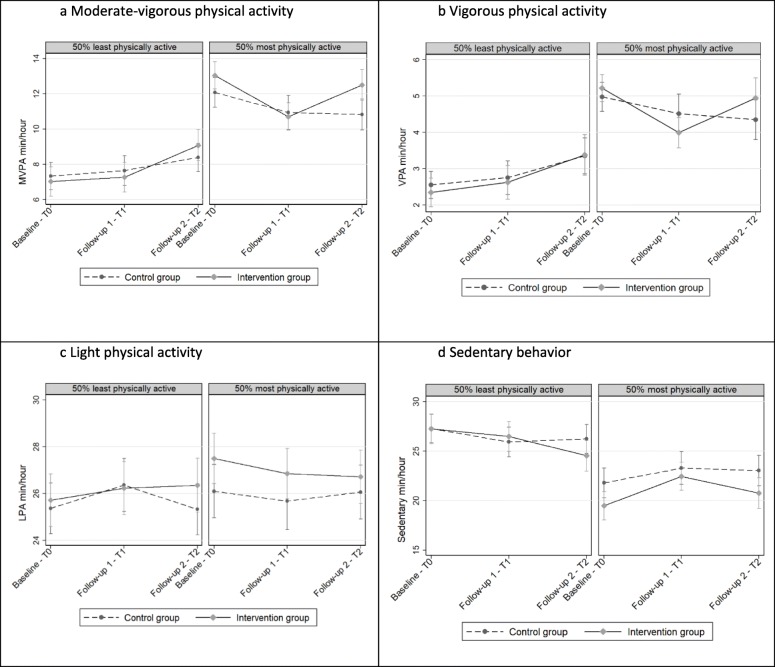
**a–d** Intervention effect on MVPA and sedentary behavior min/hour by activity level least vs most active. T0: *N* = 426, T1: *N* = 383, T2: *N* = 313

Among the 50% least physically active children at baseline, MVPA min/hour from baseline to follow-up 2 increased by 0.99 MVPA min/hour (95% CI -0.09: 2.06) in the intervention group in comparison to the control group. There was no significant difference between the intervention group and the control group among the 50% most physically active children in terms of change from baseline to follow-up 2 (Fig. 3). In the 50% least physically active children, we observed a significant negative change in sedentary behavior min/hour (− 1.67 min/hour (95% CI -3.12: − 0.21) between baseline and follow-up 2, in the intervention group compared to the control group (Fig. 3d). We did not observe a similar difference between the intervention and control group in the 50% most physically active children.

## Discussion

The present study evaluated the effects of the *Active Play in ASP* intervention on the PA level and sedentary behavior time among first graders in Norwegian ASPs. As far as we know, this is the first systematically developed, theory-based PA intervention study carried out in a Scandinavian ASP setting. Overall, the intervention did not result in significant short-term or long-term increases in the mean MVPA, VPA, or LPA among the intervention group participants in comparison to control group participants—nor did the intervention group decrease their average sedentary behavior time in comparison to the control group. However, exploratory analyses showed significant long-term effect of the intervention on sedentary behavior time in children who were among the least active 50% at baseline.

Although not significant, there was a difference in time spent in MVPA at baseline in favor of the intervention ASPs. The results showed a non-significant decrease in the mean MVPA and VPA and an increase in the sedentary time in the intervention group at follow-up 1. One year later, the levels of MVPA, VPA, and sedentary time in the intervention group were approximately equal to baseline. One explanation for this finding is that we experienced a Hawthorne effect [34]—the staff in the intervention ASPs knew that they were about to enter a PA intervention and thus made extra efforts to engage the children in PA at the beginning of the study. One way to avoid this would be to randomize ASPs following the baseline measurements. Such a strategy was not possible due to practicalities and time restrictions.

There are other possible explanations for the lack of an overall effect related to the *Active Play in ASP* intervention content and structure as well as to the intervention fidelity. The intervention built on prior research by answering a call for competence building among ASP staff in order to increase and support PA and physical activity play among children. The 7-month intervention was, hence, directed at the staff by aiming to enhance their knowledge about children’s activity play and to develop their PA-supportive skills. However, the measure of the intervention effect was based on the accelerometer data obtained from children. Thus, a potential effect depended on the staff accepting and valuing the intervention content, transforming knowledge into strategies applicable for each participating ASP and implementing these strategies into everyday practice. Moreover, it also depended on whether the children “received” the intervention and whether they utilized the possibilities offered through the physical environment—such as accessing new outdoor areas, playing in the school gym, and hiking in the neighborhood. One might argue that a more standardized and instructive PA program could have increased the likelihood—at least for a short-term effect. However, during the developmental process, our co-partners in the field emphasized the importance of designing an adaptable and flexible intervention [22]. Based on previous research, we knew that highly structured and standardized programs have limited long-term effects on children’s PA levels [35]. Moreover, such programs would have conflicted with our belief in the value of providing environmental support of children’s self-managed activity play [12, 36–39]. Putting all these considerations into practice certainly ensured the autonomy of the participating ASPs but it gave us less control over the implementation of intervention strategies. The course program highlighted the importance of organizational efforts for promoting PA among all children in the ASP and encouraged the intervention ASPs to provide children with opportunities for increased PA both indoors and outdoors. However, the very idea of ASPs in Norway is based on the notion that ASP time is the children’s leisure time during which they can, to a large extent, decide what to do on their own [16, 17]. Changing practice takes time, both on a personal and an institutional level, and 7 months may have been too short a time span for individual staff members to achieve both the intuition and didactical orientation toward becoming a more activity-supportive adult. The increased long-term results may be a reflection of a process that continued after the intervention ended.

Children spend a limited amount of time in the ASP and the narrow 2–3 h window makes it challenging to intervene and track changes properly. Thus, another probable cause for the lack of an overall effect on PA levels is a ceiling effect—at least among the most active children. On average, at the beginning of the study, the children accumulated almost 50% of the recommended 60 min of daily MVPA while being in the ASP [13]. The proportion of MVPA during ASP-time is equal to or above similar studies [18, 19]. There is a threshold as to how much MVPA a child can produce within the 2–3 h that they spend in the ASP each day, when time also has to be devoted to meals and other more stationary activities. Thus, on average, there may not be a lot more to be gained within the ASP opening hours. The decline in PA by age is found to correspond with start of school [40]. However, the no-decline in both groups in the present study suggests that the ASP is an area of relatively stable PA-levels. Our previously published baseline analysis showed that the variation in the MVPA levels was large among the participating children [13]. Following the assumption that there is a limit to how much MVPA a child can engage in during ASP time, there is great value in supporting PA among the least active children in order to help them maximize their potential.

At baseline, both gender and BMI were associated with MVPA, where girls performed less MVPA in comparison to boys and children who were either overweight or obese performed less MVPA in comparison those with normal weight [13]. *Active Play in ASP* did not significantly increase MVPA among girls nor overweight/obese children. However, while we observed no difference between the intervention and control groups in MVPA change from baseline to follow-up 2 among the most active children, we observed a borderline statistically significant increase in MVPA (0.99 min per hour) in the intervention group relative to the control group among the least physically active children. We also found that the least active 50% in the intervention group decreased their sedentary behavior time significantly (1.67 min/hour) contrasted with the control group. Assuming that a child spends 2.5 h in the ASP a day, this accumulates to 4.2 min of less sedentary time, which is an improvement in a positive direction. During 1 week, this would add up to over 20 min. If these minutes are spent in light, moderate, or vigorous PA, they would positively contribute to health and the development of motor skill competencies. It has been shown that replacing 10 min of daily sedentary behavior with MVPA and LPA improves the cardiometabolic risk profile for young people [41].

PA recommendations put great emphasis on the health and well-being benefits of MVPA [1, 42]. This, combined with the development of usable and validated accelerometers that makes it possible to measure PA intensity in large populations, may explain the emphasis placed on the mapping of MVPA in children’s and young people’s lives. However, the reciprocal and dynamic relationship between motor competence and PA requires activities at a wide range of intensity levels, including LPA. Research has shown that activity recorded by accelerometers as “light” could include physical activities important for motor development—e.g., climbing and balancing [10]. However, accelerometer data alone cannot provide information on such activity types. In an upcoming article, we present data from the current sample, mixing objectively measured PA with direct observation to capture and describe the PA variability within the entire PA spectrum.

*Active Play in ASP* was not aimed at any specific subgroup of children. On the contrary, it was directed at how the ASP staff could support PA among all children. It is possible that the least active children would have profited more if the course program had an even stronger emphasis on how to identify and support activity among children who do not spontaneously choose to partake in vigorous activities when they are given an option. More frequent and closer follow-up periods and involvement by school physiotherapists is one possible strategy. In addition, research has found that sedentary behaviors are a distinct group of behaviors that may require specific means in order to be reduced [43]. Consequently, a more explicit focus on strategies to limit sedentary time in addition to approaches to increase PA could have benefitted the least active children even more. The upcoming process evaluation study will hopefully provide answers to why the intervention did not result in an increase among the least active children and if there may be alternative strategies that are more profitable to reach this group.

### Limitations

A limitation of this study was its relatively low number of clusters included. Moreover, we have no information about the children whose parents did not accept the invitation for participation or whether the participants were representative of pupils that attend ASPs in Norway, which has implications with regards to the generalizability of the results. We measured PA during one chosen week at each timepoint. The level of PA among the children may have been affected by specific activities taking place during that exact week. Further, as we measured PA during ASP opening hours only, we have no information on whether the intervention impacted the overall PA-levels of the children. It must also be considered that there is no unified agreement on MVPA cut points for children. We used a frequently applied and recommended but relatively high cut [25, 26], meaning that minutes spent in MVPA would be even higher had we applied a lower cut. Another limitation with using the accelerometer measurement relates to the loss of information on isometric moderate and vigorous activities such as bike cycling. Large tricycles are often used standard equipment in most ASPs.

## Conclusion

The *Active Play in ASP* intervention was not effective at increasing the mean PA nor in reducing sedentary behavior among the participating children compared to controls; however, an exploratory analysis indicated a minor long-term effect on the reduction of sedentary behavior time among the least active 50% of the children—which is an improvement in a positive direction. It is possible that an intervention that was more specifically targeted at children who engage less in PA and physical activity play would have been even more beneficial. Engagement in PA requires motor skill competence, while development of motor skill competence depends on a variety of activity types that contain a range of intensity levels. A more explicit focus on strategies to reduce sedentary behavior would potentially increase the time spent on motor competence building activities as well as more vigorous PA. This may require a closer and more frequent follow-up by experts. Additional research should build on our experiences and investigate strategies to strengthen the skills of the ASP staff needed to identify and engage the less physically active children in an ASP setting.

## Data Availability

The current study has been approved by the Norwegian Data Protection Official for research related to the specific aim of the project. Thus, the data analyzed are not publicly available.

## Abbreviations

PA: physical activity
ASP: after-school program
MVPA: moderate to vigorous physical activity
LPA: light physical activity
VPA: vigorous physical activity
PT: physiotherapist
PE: physical education
CPM: counts per minute
BMI: body mass index
LDA: longitudinal data analysis
MICE: multiple imputation by chained equations
